# A case series study of compassion‐focused therapy for distressing experiences in psychosis

**DOI:** 10.1111/bjc.12437

**Published:** 2023-08-27

**Authors:** Charles Heriot‐Maitland, Andrew Gumley, Til Wykes, Eleanor Longden, Chris Irons, Paul Gilbert, Emmanuelle Peters

**Affiliations:** ^1^ Department of Psychology, Institute of Psychiatry, Psychology and Neuroscience King's College London London UK; ^2^ Glasgow Mental Health Research Facility University of Glasgow Glasgow UK; ^3^ Psychosis Research Unit Greater Manchester Mental Health NHS Foundation Trust Manchester UK; ^4^ Division of Psychology and Mental Health The University of Manchester Manchester UK; ^5^ Complex Trauma and Resilience Research Unit Greater Manchester Mental Health NHS Foundation Trust Manchester UK; ^6^ Balanced Minds London UK; ^7^ Centre for Compassion Research and Training, College of Health, Psychology and Social Care University of Derby Derby UK; ^8^ The Compassionate Mind Foundation Derby UK; ^9^ South London and Maudsley NHS Foundation Trust London UK

**Keywords:** auditory hallucinations, case series, compassion‐focused therapy, delusions, psychosis, voices

## Abstract

**Objectives:**

Compassion‐focused therapy (CFT) is an evolution‐informed biopsychosocial approach that seeks to cultivate attachment and care motivational systems and their psychophysiological regulators. These can counteract some of the harmful effects of social threat, inferiority, shame, self‐criticism and depression, which are common in people with psychosis and undermine their well‐being, social trust and ability to feel safe. This study aimed to test the acceptability of a novel manualized individual CFT intervention for psychosis (CFTp).

**Design:**

A non‐concurrent, multiple‐baseline, case series design, with three phases: baseline, intervention and follow‐up.

**Methods:**

The 26‐session CFTp intervention was provided for a sample of eight people with distressing psychotic experiences and a psychosis‐related diagnosis. The study aimed to assess acceptability of CFTp and to test clinically reliable improvements while receiving the intervention, compared to a baseline period.

**Results:**

Seven of eight participants completed the therapy, and clinically reliable improvements were found at both the single‐case and group level of analysis. At the single‐case level, over half the participants showed improvements in depression (5/7), stress (5/7), distress (5/7), anxiety (4/7) and voices (3/5). One participant showed a deterioration in anxiety (1/7) and dissociation (1/7). At the group level (*n* = 7), there were significant improvements in depression, stress, distress, voices and delusions. The improvements in voices, delusions and distress were sustained at 6‐ to 8‐week follow‐up, but depression and stress dropped slightly to trend‐level improvements.

**Conclusions:**

CFTp is a feasible and acceptable intervention for psychosis, and further investigation is warranted with a randomized controlled trial.

**Practitioner points**

- CFTp is acceptable for people with distressing voices and delusions in psychosis
- CFTp can successfully target social‐rank processes such as self‐criticism and shame
- Improved outcomes are reported in depression, stress, distress, voices and delusions
- CFTp can be delivered as a manualized intervention, suitable for RCT evaluation

## INTRODUCTION

Compassion‐focused therapy (CFT) was developed for helping individuals with complex and enduring mental health difficulties, particularly those involving complex forms of shame and hostile forms of self‐criticism (Gilbert, 2009; Gilbert & Simos, 2022). People with mental health issues often get caught in, and struggle with, difficulties in competitive and social‐ranking motive systems: they are threat‐focused; sensitized to social conflict, shame and rejection; experience hostile forms of self‐criticism and find it difficult to be caring and supportive of themselves. People with psychosis often experience hostile, condemning and threatening voices [in contrast to the benevolent voices heard by healthy voice‐hearers (Baumeister et al., 2017)] which can reflect how individuals experience others (Birchwood et al., 2000, 2004, 2007, 2014; Gilbert et al., 2001). Rather than trying to modify processes within the social competitive and ranking motivation systems, CFT helps people switch into a different motivational system, that is, moving out of social‐ranking ‘mentalities’ that focus attention on the (potentially harmful) power of others, towards activating cooperative, caring and affiliative processes to self and others. There is evidence that training compassionate practices, intentions and skills can impact a range of neural circuits, immune system and the autonomic nervous system (Singer & Engert, 2019; Weng et al., 2013, 2018).

There is growing evidence for the effectiveness of CFT in targeting these processes across different clinical populations (Millard et al., 2023), for example, complex difficulties (Gilbert & Procter, 2006), personality disorders (Lucre & Corten, 2013), eating disorders (Gale et al., 2014) and bipolar affective disorder (Gilbert et al., 2022). Theoretical reviews by Gumley et al. (2010), Heriot‐Maitland et al. (2019) and Heriot‐Maitland (2023) suggest that CFT is ideally suited to address the kinds of threat‐based difficulties experienced by people with psychosis.

CFT with a psychosis population has been investigated in a handful of small studies, which showed promising indicators. In a case series of Compassionate Mind Training (the practices employed in CFT) for people who hear malevolent voices (Mayhew & Gilbert, 2008), participants used ‘compassionate self’ imagery to develop empathy for their distress and self‐criticism. They found that working with self‐critical thoughts (not critical voices directly) led to a reduction in the malevolence of voices. Braehler et al. (2013) delivered CFT groups involving practice and skills in compassion applied to shame, stigma, paranoia, self‐attacking and hostile voices. Participants showed significant reductions in depression associated with psychosis, reductions not seen in the control group.

Specific CFT techniques have also been tested in clinical populations with paranoid beliefs. Ascone et al. (2017) tested a brief (one session) compassion‐focused imagery intervention, while Forkert et al. (2022) explored the feasibility of brief (four sessions) compassionate imagery intervention that involved developing a ‘compassionate coach’. Both studies reported good acceptability of techniques, but only one (Forkert et al., 2022) demonstrated improvement in paranoia, which may be due to the slightly longer intervention used. Similar imagery‐based interventions for paranoia have been tested in larger studies with non‐clinical populations. Lincoln et al. (2012) found that, compared to the control group, participants receiving compassion‐focused imagery had lower negative emotion, higher self‐esteem and less paranoid thoughts. Brown et al. (2020) tested compassionate imagery with the aid of virtual reality scenarios. They found that, compared to controls, paranoia levels decreased significantly among participants who practiced the ‘compassionate coach’ technique (study 1) and in those who practiced ‘loving kindness meditation’ (study 2).

Finally, there have been several single‐case studies of CFT for psychosis (Ellerby, 2013a, 2013b, 2014a, 2014b; Heriot‐Maitland & Levey, 2021; Heriot‐Maitland & Russell, 2018). These involved individual CFT formulations and longer‐term interventions utilizing multiple CFT techniques. These reports provide helpful indicators of the relevance and acceptability of using CFT formulations to guide and tailor therapy processes for individuals with distressing voice‐hearing and delusions. However, to advance CFTp research, manualized interventions are required for developing larger‐scale effectiveness studies, to standardize the intervention across multiple participants and therapists.

### Summary and aims

This study aimed to develop and test the acceptability of a manualized individual CFTp intervention. It also investigated the effects of CFTp and processes of change. The CFT model predicts that for people with psychosis, threat‐based dissociative and psychotic processes would be accentuated by social‐ranking signals (such as shame and self‐criticism) and attenuated by communication signals linked to caring, support and social safeness (such as compassion to/from self and others; Heriot‐Maitland et al., 2019). The CFTp intervention, therefore, aimed to target social‐rank threat by helping people to build social safeness and to develop compassionate motives to themselves, others and their distressing experiences.

### Research questions and hypotheses

Is CFTp feasible and acceptable?

Can CFTp make targeted changes to key social processes (social safeness, compassion and social‐rank threat), with the desired effect on outcomes (psychotic symptoms, dissociation and depression)?

The following hypotheses were made:

1. Significant changes in outcome and process measures will occur during the intervention, and not the baseline, phase;
2. Significant changes in process measures will precede changes in outcome measures;
3. Significant changes in outcome and process measures will remain 6–8 weeks post‐intervention;
4. Session‐by‐session measures will improve significantly in the intervention, compared to the baseline, phase.

## METHODS

### Design and participants

A non‐concurrent multiple‐baseline, single‐case experimental design (Watson & Workman, 1981) was used, with three phases: baseline, intervention and follow‐up. *Baseline phase*: Participants were randomly allocated to one of three baseline periods (2, 4 and 6 weeks), before commencing the intervention. *Intervention phase*: 26 weekly sessions over 6–9 months. *Follow‐up phase*: 6–8 weeks after finishing the intervention.

Participants were secondary care mental health service users with distressing psychotic experiences and a psychosis‐related diagnosis, recruited from two sites: one in London, United Kingdom (South London and Maudsley NHS Foundation Trust), and one in Glasgow, United Kingdom (NHS Greater Glasgow and Clyde). The inclusion criteria were: (i) ≥18 years old; (ii) psychosis‐related diagnosis (F20‐39); (iii) distressing positive symptoms (voices and/or delusions), scoring 2+ on ‘intensity of distress’ item of the Psychotic Symptoms Rating Scales (PSYRATS; Haddock et al., 1999); and (iv) not currently engaged in, or having completed 12+ sessions of, psychological therapy within the last 3 years.

### Measures

#### Outcome measures

*Psychotic symptoms* were measured by the *PSYRATS* (Haddock et al., 1999), a 17‐item interview‐rated scale measuring hallucinations (11 items) and delusions (six items). Items are rated 0–4, with total range 0–44 (hallucinations) and 0–24 (delusions). PSYRATS has high inter‐rater reliability and was designed to be sensitive to change with psychological interventions for psychosis.

*Depression and anxiety symptoms* were measured by the *Depression, Anxiety and Stress Scale* (*DASS‐21*; Lovibond & Lovibond, 1995), a 21‐item self‐report measure. Items are rated on a four‐point scale (0 = never to 3 = almost always) relating to the past week. Totals range 0–42 (depression), 0–42 (anxiety) and 0–42 (stress). The subscales have good internal consistency, including in a psychosis sample (Cronbach's alpha: .93 depression, .91 anxiety and .93 stress; Huppert et al., 2002).

*Psychological distress* was measured by the *Clinical Outcomes in Routine Evaluation* (*CORE*; Barkham et al., 2009), a 34‐item self‐report scale measuring subjective well‐being, symptoms, functioning and risk/harm. Total mean scores range 0–4, and the scale has good internal consistency (Cronbach's alpha .94).

*Dissociation symptoms* were measured by the *Revised, Dissociative Experiences Scale* (*DES‐II*; Carlson & Putnam, 1993), a 28‐item self‐report scale. Items are rated as percentages (in 10% increments from 0% = never to 100% = always), with scores converted to a 0–10 rating, total range 0–280 (Cronbach's alpha .90). This study used a state‐adapted version, asking specifically about ‘the past week’.

#### Process measures

*Social‐rank threat, safeness and compassion* were measured by: (i) *Social Comparison Scale* (*SocCS*; Allan & Gilbert, 1995), an 11‐item self‐report scale (rating 1–10, total score range 11–110; Cronbach's alpha .91) measuring social‐rank and relative social standing; (ii) *Forms of Self‐Criticizing/Attacking and Self‐Reassuring Scale* (*FSCSR*; Gilbert et al., 2004), a 22‐item self‐report scale (rating 0–4) measuring self‐criticism (‘inadequate self’ nine items, range 0–36, Cronbach's alpha .90; ‘hated self’ five items, range 0–20, Cronbach's alpha .86; ‘self‐reassurance’ eight items, range 0–32), Cronbach's alpha .86; (iii) *Other as Shamer Scale* (*OAS*; Goss et al., 1994), an 18‐item self‐report measure of external shame (rating 0–4, range 0–72; Cronbach's alpha .92); (iv) *Self‐Compassion Scale—Short Form* (*SCS‐SF*; Raes et al., 2011), a 12 item self‐rated scale measuring self‐compassion (rating 1–5, range 12–60; Cronbach's alpha .86); and (v) *Personal Beliefs about Illness Questionnaire*—*Revised* (*PBIQ‐R*; Birchwood et al., 2012), a 20‐item self‐report measure (rating 1–4, range 20–80) of social‐rank variables in psychosis, with five subscales: shame, loss, entrapment, control over illness and social marginalization/group fit. Cronbach's alpha values for the subscales are adequate, ranging from .72 to .81.

*Heart rate variability* (HRV) was measured at the start of each assessment using a single‐channel ECG waveform recorder (Actiwave Cardio, CamNtech Ltd.) connected to the chest by two ECG pads. For 5 min, a resting HRV recording was taken as participants sat normally in a chair, and a further 2‐min recording as participants engaged with a breathing exercise, using an app (Breathing Zone). The measurement used was the RMSSD (Root Mean Square of Successive Differences in the inter‐beat intervals). RMSSD is recommended for the time‐domain assessment of HRV (Malik et al., 1996), and increases in RMSSD scores signal improvements in HRV, which is indicative of improved parasympathetic regulatory influence on the heart.

#### Session‐by‐session measures

*Social safeness* was measured by the *Social Safeness and Pleasure Scale* (*SSPS*; Gilbert et al., 2009), an 11‐item self‐report scale measuring the extent to which people experience their social worlds as safe. Items are rated on a five‐point scale (1 = almost never to 5 = almost all the time, range 11–55). The scale has high internal consistency (Cronbach's alpha .92).

*Dissociation* was measured by a three‐item self‐report scale (1 = not at all to 7 = very much, range 3–21), consisting of: ‘since the last session I've found it difficult to focus on what was happening around me’, ‘since the last session I've been easily distracted’ and ‘since the last session I've found myself doing things without paying attention’. These items were validated against the *DES‐II* and used in a previous study to measure fluctuations in dissociation (Varese et al., 2011).

### Procedure

The study received ethical approval from the London Dulwich Research Ethics Committee (REC reference 15/LO/0198), and Research and Development approval from the two NHS sites in London and Glasgow. Participants were identified and invited to participate by their clinical teams. Those interested were invited to meet with the researcher to discuss the study and provide written consent.

The study involved five data collection points: T1 (start‐baseline); T2 (end‐baseline); T3 (mid‐therapy); T4 (end‐therapy); T5 (follow‐up). Each assessment lasted around 1 hr. Brief sessional measures were also administered every week (during baseline and intervention, but not follow‐up phase). During the intervention phase, these were administered at the start of sessions. Most measures were self‐report questionnaires, answered electronically by participants on a laptop. The only two measures that required researcher involvement were PSYRATS and HRV. PSYRATS was scored by the lead researcher (CHM), and HRV was measured with assistance from research nurses from both the NIHR/Wellcome Trust King's Clinical Research Facility at King's College Hospital and the Glasgow Clinical Research Facility. Participants received an honorarium and travel expenses at each assessment. They were asked for their consent to record therapy sessions (audio, video or both) for clinical supervision and fidelity‐checking purposes.

### Intervention

The intervention was provided by a clinical psychologist and developer of the CFTp intervention (author CHM), with adherence to a 50‐page manual developed in collaboration with CFT experts and experts by experience. Table 1 shows the Template for Intervention Description and Replication (TIDieR) checklist (Hoffmann et al., 2014). The content page of the CFTp manual is provided in Supporting Information (Table S1), and the full manual is available from the first author on request.

**TABLE 1:** Template for intervention description and replication (TIDieR) checklist.

1. Brief name	Compassion‐focused therapy for psychosis (CFTp)
2. Why	To target changes to key social processes (i.e., increasing social safeness, compassion and decreasing social‐rank threat), with an effect on clinical outcomes (i.e., reducing psychotic symptoms, dissociation and depression)
3. What (materials)	A CFTp manual
4. What (procedure)	The CFTp manual is divided into the following levels: *Starting therapy* Establishing safeness and connection Learning about evolved (tricky) brains, emotional systems and multiple selves Understanding how my emotions and mind have become shaped Building the compassionate self Directing compassion to self, others, emotional parts and voices *Ending therapy* Full descriptions and guidance for each level are provided in the manual
5. Who provided	Clinical psychologist with 10+ years' experience working with people with psychosis, with monthly supervision from a CFT expert. As the therapist was the developer of the intervention, there was no specific training in CFT for psychosis; however, the therapist had trained in (generic) CFT, at both the introductory and advanced levels
6. How	Face‐to‐face sessions, individually
7. Where	NHS therapy/consultation room
8. When and how much	26 × 1‐hr weekly sessions, over 6–9 months
9. Tailoring	The CFTp manual includes the following instructions for tailoring: ‘The ordering of 1–5 levels is to guide the therapist through the therapy content. In reality this is a process‐driven therapy, so therapists will be following the client, using clinical judgment, supervision and collaborative discussion to transition to/from each level. This may involve re‐ordering levels 1–5, blending one with another, or skipping one out completely. The phrases [described in the manual] are also to guide, rather than to quote. The therapist will be talking to clients in their own language, at their own pace, using Socratic questioning, and attending to process’. (CFTp manual, 2018; contents page in Table S3)
10. Modification	Modifications were made to the manual over the intervention period (2015–2018), as more was learnt about applying CFT with this population. Full details of the modifications made between the CFTp manual version 1 (December 2014) and CFTp manual version 2 (December 2018) are reported in Heriot‐Maitland (2020) pp. 103–123
11. How well (planned)	A CFTp Adherence and Competence Measure (CFTp‐ACM) was developed to assess fidelity to the manual and therapist competence in CFT. In the CFTp‐ACM, there are 21 manual adherence items and 23 therapist competence items. All are rated on a five‐point scale from 0 (‘absent or inappropriate’) to 4 (‘skilful enactment’). The 21 adherence items relate to the key elements in the manual, which had been developed specifically for the current (psychosis) population, and the 23 competence items were taken from an existing scale that had been developed for generic CFT research, namely the *Compassion Focused Therapy Therapist Rating Scale* (*CFT‐TCRS*; Horwood et al., 2019). The 23‐item CFT‐TCRS is comprised of 14 unique competencies (e.g., ‘compassionate mind training’ and ‘multiple selves’) and nine microskills (e.g., ‘pacing’ and ‘mentalization’). The CFTp‐ACM is available from authors on request
12. How well (actual)	Five audios were sent to an external CFTp expert for fidelity checking against the CFTp‐ACM. To ensure that fidelity‐checking procedures covered a suitable breadth of different therapy levels, and a suitable breadth of participants in the sample, audios were selected so that each of the five therapy levels was represented by a different participant. The adherence ratings averaged 3.86 (on the scale 0 ‘absent or inappropriate’ to 4 ‘skilful enactment’), and the therapist competence ratings averaged 3.67 on the same scale. For the two subscales within the therapist competence scale, the ratings averaged 3.80 for CFT unique competencies and 3.64 for CFT microskills. Overall, the therapist was rated as ‘excellent’ as a CFT therapist for three of the sessions, ‘very good’ for one session and ‘good’ for one session. These ratings confirmed that the therapist in this study was adherent to the CFTp manual and competent in providing the CFTp intervention

### Data analysis

The primary research question (feasibility and acceptability) was investigated by a CONSORT diagram of recruitment and attrition.

To test hypotheses 1–3, the *single‐case‐level* analysis used a Reliable Change Index (RCI; Jacobson & Truax, 1991) to determine which changes were greater than would be expected from the standard variability of measures. In line with the internal consistency method for clinical populations (Martinovich et al., 1996), the RCI analysis required reliability (internal consistency) and standard deviations of each measure, which were sourced from existing normative data. The RCI is computed by dividing the change scores by the standard error of change between scores. If the RCI is >1.96, the change is considered to be reliable, rather than a result of measurement error (Jacobson & Truax, 1991).

The *group‐level* analysis used a Wilcoxon signed‐rank test to compare changes in group means across phases. For hypothesis 1, the comparison was between ‘baseline’ (T1 → T2) and ‘intervention’ (T2 → T4) phases. For hypothesis 2, the mid‐therapy timepoint (T3) enabled comparisons between ‘first half’ (T2 → T3) and ‘intervention’ (T2 → T4). For hypothesis 3, the follow‐up timepoint (T5) was used to compare between ‘baseline’ (T1 → T2) and ‘intervention + follow‐up’ (T2 → T5) phases.

To test hypothesis 4, the *single‐case‐level* analysis used a Tau‐U statistic to test for significant differences between baseline and intervention phases (i.e., a significant degree of non‐overlap between the two phases; Parker et al., 2011). At the *group level*, Tau‐U scores for each participant were combined to produce a Tau‐U Omnibus score.

## RESULTS

### Feasibility and acceptability

**FIGURE 1:**
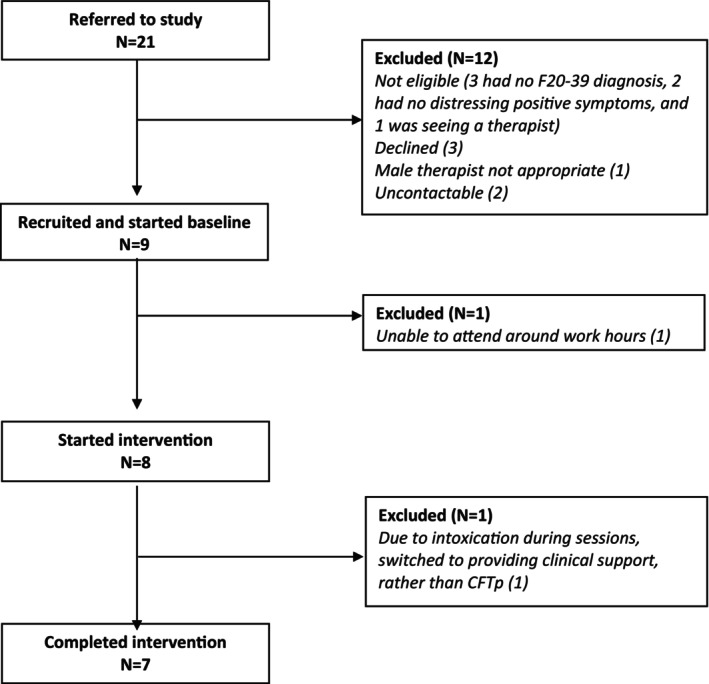
Flow diagram of participant recruitment.

The CONSORT (Figure 1) shows participant recruitment and attrition flow. Of the 21 referrals, nine (43%) were eligible and willing to participate in the study, eight started the intervention (one did not start due to work commitments), and seven completed it. Table 2 shows clinical and demographic information for the final sample of seven, including age, sex, ethnicity and diagnosis. One participant had distressing voices only, two had distressing delusions only, and four had distressing voices and delusions. Information on the history of psychosis was not known for one participant, but for the remaining six, the majority had experienced psychosis for 13–15 years prior to the start of therapy (sample mean 16 years).

**TABLE 2:** Clinical and demographic profiles of participants (*n* = 7).

Participant	Age	Sex	Site	Ethnicity	Diagnosis	Years with psychosis	Current positive symptoms	
							Voices (*distress*)	Delusions (*distress*)
P1 Steve^a^	53	Male	London	White British	F21	13	Yes (*3 marked*)	Yes (*3 marked*)
P2 Greg	64	Male	London	White British	F29	Unknown	Yes (*4 extreme*)	Yes (*4 extreme*)
P3 Thomas	37	Male	London	White Irish	F29	2	Yes (*4 extreme*)	Yes (*4 extreme*)
P4 Tosin	48	Male	London	Black British	F32	15	No	Yes (*3 marked*)
P5 Charmaine	58	Female	London	Black British	F32	15	Yes (*4 extreme*)	No
P6 Amanda	36	Female	Glasgow	White British	F20	15	Yes (*2 moderate*)	Yes (*2 moderate*)
P7 Gareth	57	Male	Glasgow	White British	F20	34	No	Yes (*3 marked*)

^a^ Names have been changed.

The only non‐completer finished at the mid‐therapy point (13 sessions) due to a relapse in heavy alcohol use from session 7 or 8 onwards. Clinical support continued for this individual, but because of high levels of intoxication during sessions, it was not possible to continue CFTp, and therefore, the data could not be used. Alcohol abuse had been a chronic pre‐existing condition for this participant but had not been a problem at recruitment. This is discussed further in the discussion.

All seven provided full outcome and process measures across all assessment points, demonstrating feasibility and acceptability of research procedures. This included HRV readings, although some had to be excluded due to incorrect software set‐up (four of the 35 readings) and poor ECG connection (four of 35), which were later discovered when processing the data. The missing HRV data were due to technical issues, rather than participant acceptability or willingness to provide data.

Only one participant (P1) did not consent to audio‐recording (for paranoia‐related concerns), but none opted for video‐recording. The session‐by‐session measures were acceptable for six of the participants for the duration of therapy. One participant (P1) provided weekly measures initially but asked to stop after 10 sessions due to finding them irritating and confusing.

### Changes in process and outcome measures

Hypothesis 1 Significant changes in outcome and process measures will occur during the intervention, and not the baseline, phase.

*Single‐case‐level* analysis (RCI) summary results are shown in Table 3. (RCI results for each individual case are in Tables S2 and S3). With process measures, there were reliable improvers on PBIQ‐R (6/7), FSCSR‐Hate (5/7), SocC (4/7), FSCSR‐Inad (4/6) and SCS‐SF (4/7). Improvements were more so in the intervention than baseline phase.

**TABLE 3:** Directions of reliable changes in measures, showing only those that were significant against a RCI.

Summary of all seven cases									
	T1 → T2	T2 → T3	T2 → T4	T2 → T5		T1 → T2	T2 → T3	T2 → T4	T2 → T5
*Process measures (RCI)*					*Outcome measures (RCI)*				
SocC (*n* = 7)	↓ 2	**↑ 3**	**↑ 4**	**↑ 4**	PSYRATS‐V (*n* = 5)			**↓ 3**	**↓ 4**
FSCSR‐Inad (*n* = 7)	**↓ 1**	**↓ 2**	**↓ 4**	**↓ 5**	PSYRATS‐D (*n* = 6)		**↓ 1**	**↓ 2**	**↓ 1**
FSCSR‐Reas (*n* = 7)	**↑ 1**↓ 1	**↑ 3**↓ 2	**↑ 3**	**↑ 2**↓ 1	DASS‐Dep (*n* = 7)	**↓ 1**	↑ 1**↓ 5**	**↓ 5**	**↓ 5**
FSCSR‐Hate (*n* = 7)	↑ 1	**↓ 5**	**↓ 5**	**↓ 4**	DASS‐Anx (*n* = 7)	↑ 1 **↓ 2**	↑ 1 **↓ 4**	↑ 1 **↓ 4**	↑ 2 **↓ 3**
OAS (*n* = 7)	**↓ 2**	**↓ 3**	**↓ 3**	**↓ 5**	DASS‐Str (*n* = 7)	↑ 2 **↓ 1**	↑ 1 **↓ 5**	**↓ 5**	↑ 1 **↓ 4**
SCS‐SF (*n* = 7)	**↑ 1**	**↑ 1** ↓ 1	**↑ 4**	**↑ 3**	CORE (*n* = 7)	↑ 1	**↓ 4**	**↓ 5**	**↓ 5**
PBIQ‐R (*n* = 7)		**↓ 6**	**↓ 6**	**↓ 7**	DES‐II (*n* = 7)		**↓ 3**	↑ 1 **↓ 2**	**↓ 4**
RMSSD (ms) (*n* = variable)	**↑ 1** ↓ 1 (*n* = 4)	**↑ 2** ↓ 1 (*n* = 6)	**↑ 2** (*n* = 5)	↓ 1 (*n* = 5)					

*Note*: Bold indicates number of cases in direction of improvement; non‐bold indicates number in direction of deterioration.

Abbreviations: CORE, Clinical Outcomes in Routine Evaluation; DASS, Depression Anxiety and Stress Scales (Dep = Depression, Anx = Anxiety, Str = Stress); DES‐II, Revised, Dissociative Experiences Scale; FSCSR, Forms of Self‐Criticizing/Attacking and Self‐Reassuring Scale (Inad = Inadequate self, Reas = Self‐reassurance, Hate = Hated self); OAS, Other as Shamer Scale; PBIQ‐R, Personal Beliefs about Illness Questionnaire—Revised; PSYRATS, Psychotic Symptoms Ratings Scales (V = Voices, D = Delusions); RMSSD (ms), Root Mean Square of Successive Differences (in milliseconds); SocC, Social Comparison Scale; SCS‐SF, Self‐Compassion Scale—Short Form.

With the outcome measures, there were reliable improvers on DASS‐Dep (5/7), DASS‐Str (5/7), CORE (5/7), DASS‐Anx (4/7) and PSYRATS‐V (3/5). For one case (P1), two measures changed in the opposite direction (increases in DASS‐Anx and DES‐II). In the baseline phase, no one changed on three of seven outcome measures (PSYRATS‐V, PSYTRATS‐D and DES‐II) and only one or two cases for DASS‐Dep, DASS‐Anx, DASS‐Str and CORE.

*Group‐level* results are shown in Table 4. (Group means are reported in Table S4.) There were significant improvements in six process measures (SocC, FSCSR‐Inad, FSCRSR‐Hate, OAS, SCS‐SF and PBIQ‐R), none of which changed during baseline. Only two process measures did not change significantly (FSCSR‐Reas and RMSSD), although both improved at trend level (*p* = .072 and *p* = .080).

**TABLE 4:** Wilcoxon signed‐rank tests for group‐level changes across phases.

	Baseline phase (T1 → T2)		1st half phase (T2 → T3)		Intervention phase (T2 → T4)		Follow‐up (T2 → T5)	
	*Z*	Sig	*Z*	Sig	*Z*	Sig	*Z*	Sig
Outcome measures								
PSYRATS‐V	−.378	.705	−2.032	**.042**	−2.023	**.043**	−2.023	**.043**
PSYRATS‐D	−1.000	.317	−1.084	.279	−2.207	**.027**	−2.232	**.026**
DASS‐Dep	−.172	.863	−1.947	.051	−2.124	**.034**	−1.859	.063
DASS‐Anx	−.255	.799	−1.472	.141	−1.614	.106	−1.153	.249
DASS‐Str	−.412	.680	−2.032	**.042**	−2.371	**.018**	−1.876	.061
CORE	.000	1.000	−2.028	.**043**	−2.366	**.018**	−2.366	**.018**
DES‐II	−1.018	.309	−1.185	.236	−1.352	.176	−1.859	.063
Process measures								
SocC	−1.214	.225	−2.201	**.028**	−2.366	**.018**	−1.690	.091
FSCSR‐Inad	−.734	.463	−2.201	**.028**	−2.207	**.027**	−2.197	**.028**
FSCSR‐Reas	.000	1.000	−.314	.753	−2.371	.072	−.426	.670
FSCSR‐Hate	−1.625	.104	−2.371	**.018**	−2.371	**.018**	−2.201	**.028**
OAS	−.847	.397	−2.028	**.043**	−2.371	**.018**	−2.366	**.018**
SCS‐SF	−.511	.610	−.762	.446	−2.028	**.043**	−2.117	**.034**
PBIQ‐R	.000	1.000	−2.371	**.018**	−2.366	**.018**	−2.371	**.018**
RMSSD (ms)	−.365	.715	−.734	.463	−1.753	.080	−.674	.500

*Note*: Significant results in bold.

There were also significant improvements on most outcome measures (PSYRATS‐V, PSYRATS‐D, DASS‐Dep, DASS‐Str and CORE), except for DASS‐Anx and DES‐II. Again, improvements occurred in the therapy but not baseline phase.

Overall, these results provide support for hypothesis 1, at both the single case and the group levels of analysis.

Hypothesis 2 Significant changes in process measures will precede changes in outcome measures.

*Single‐case‐level* results show that a similar number of participants recorded reliable changes in the first half of therapy as in the (full) therapy. In Table 3, the only measures that show differences between ‘first half’ and ‘intervention’ are FSCSR‐Inad (3/7 to 4/7 cases), SCS‐SF (1/7 to 4/7 cases) and PSYRATS‐V (0/5 to 3/5 cases). Other outcome measures were stable in their numbers from first half to intervention.

*Group‐level* results show that most process measures that improved significantly in the (full) therapy were already improving in the first half. The exception was SCS‐SF, which only changed later in the therapy.

Among outcome measures, three improved in the first half (PSYRATS‐V, DASS‐Str and CORE), and two (PSYRATS‐D and DASS‐Dep) were slower to improve. This suggests mixed patterns of change, in that some outcomes changed concurrently with process measures (e.g., PSYRATS‐V), but others changed afterwards (PSYRATS‐D and DASS‐Dep).

Overall, hypothesis 2 was not supported at the single‐case level, but there was some evidence of process changes preceding changes in PSYRATS‐D and DASS‐Dep at the group level.

Hypothesis 3 Significant changes in outcome and process measures will remain post‐intervention.

*Single‐case‐level* results show that most improvements continued after therapy. With some measures, there was loss of one reliable improver at follow‐up, but in others, there were additional improvers; for example, OAS improved reliably for three cases in therapy (P2, P5 and P6) but increased to five by follow‐up (P1, P2, P5, P6 and P7). FSCSR‐Inad had also improved for five cases at follow‐up (up from four), and for PBIQ‐R, all seven had improved by follow‐up. An exception was RMSSD, which had two improvers in therapy (P3 and P7; of *n* = 5), but neither remained improved at follow‐up, and one (P2, who had missing data) recorded deterioration.

In terms of outcomes at follow‐up, four (of five) cases still had improved PSYRATS‐V (P2, P3, P5 and P6), and one had improved PSYRATS‐D (P2). Five had improved DASS‐Dep, four DASS‐Str and three DASS‐Anx. The only outcomes that deteriorated at follow‐up were DASS‐Anx for two cases (P1 and P7) and DASS‐Str for one (P7). Finally, five cases had improved scores on CORE, and four had improved DES‐II scores at follow‐up.

*Group‐level* results show that five of the improvements on process measures remained post‐therapy (FSCSR‐Inad, FSCSR‐Hate, OAS, SCS‐SF and PBIQ‐R), along with three of the outcome measures (PSYRATS‐V, PSYRATS‐D and CORE). The one process measure (SocC) and two outcomes (DASS‐Dep and DASS‐Str) that were no longer significant were at trend level (*p* = .091, *p* = .063 and *p* = .061, respectively). Interestingly, the DES‐II, which was not significant during therapy (*z* = −1.352, *p* = .176), continued to improve post‐therapy at trend level (*z* = −1.859, *p* = .063).

Overall, the single‐case‐level results support hypothesis 3, except for RMSSD, which had no evidence of improvements remaining after therapy. At the group level, hypothesis 3 was supported for most measures.

Hypothesis 4 Session‐by‐session measures will improve significantly in the intervention, compared to the baseline, phase.

**FIGURE 2:**
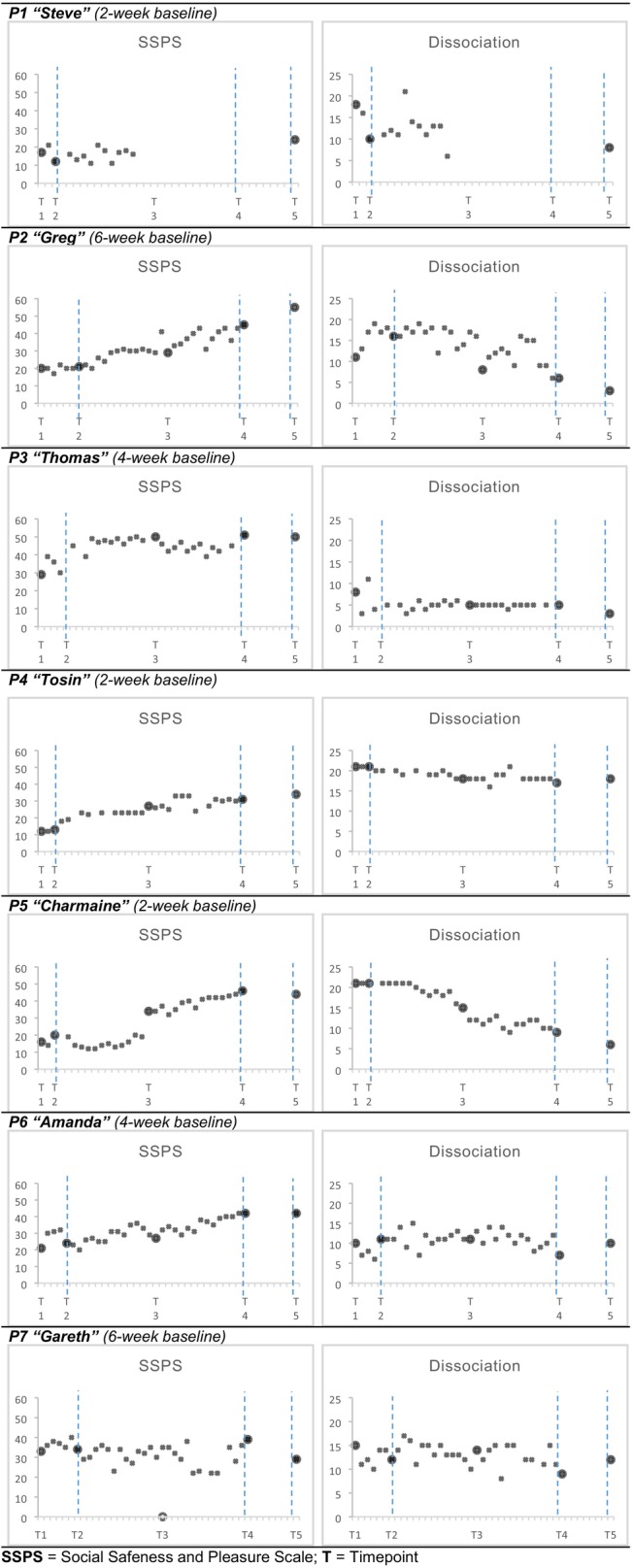
Plots of session‐by‐session measures. SSPS, Social Safeness and Pleasure Scale; *T*, timepoint.

This hypothesis was tested with visual inspection of plots (Figure 2) and with a Tau‐U analysis of degree of non‐overlap between baseline and intervention (Tables S5 and S6). One participant (P1) opted out of weekly measures, so their data were excluded from the Tau‐U analysis.

Figure 2 shows that everyone finished the study with improved SSPS and Dissociation scores. The largest SSPS changes were for cases P2 and P5; however, P5's considerable improvement over therapy (score 19 to 46) was not significant (Tau = .37, *p* = .30) because there had also been improvement in the 2‐week baseline (16 to 20). On the other hand, what looks like a very flat line for P4's Dissociation (score 20 to 18) was statistically significant (Tau = −.96, *p* < .01) due to the even flatter baseline (constant at 21, the maximum score).

Visual inspection of the two plots together shows a mirroring of trends in three or four cases. For P2 and P5, this is very clear, with Dissociation decreasing simultaneously as SSPS increases. P3 and P7 also have mirroring trends, but because the changes were smaller, this is not so apparent in the plots. P3 hit a ceiling in SSPS quickly (scoring 49 by session 4, with range 11–55), and a floor in Dissociation around the same time (scoring 3, range 3–21). While P7's scores look flat in both SSPS and Dissociation, their SSPS scores are significant in a *negative* direction (Tau = −.63, *p* = .01). This is because P7's baseline shows a steady increase in SSPS, which then stops increasing in therapy.

The cases showing significant increases in SSPS, compared to baseline, outweighed the cases who did not. This is reflected in the Tau‐U Omnibus test, where all participants are combined (Table S5). For SSPS, the Tau‐U Omnibus showed a significant upward trend (Tau = .48, *p* < .001). For Dissociation, the Tau Omnibus score showed a non‐significant downward trend (Tau = −.17, *p* = .17).

Overall, Hypothesis 4 was supported for SSPS but not for Dissociation.

### *Post‐hoc* analysis of distressing experiences

**FIGURE 3:**
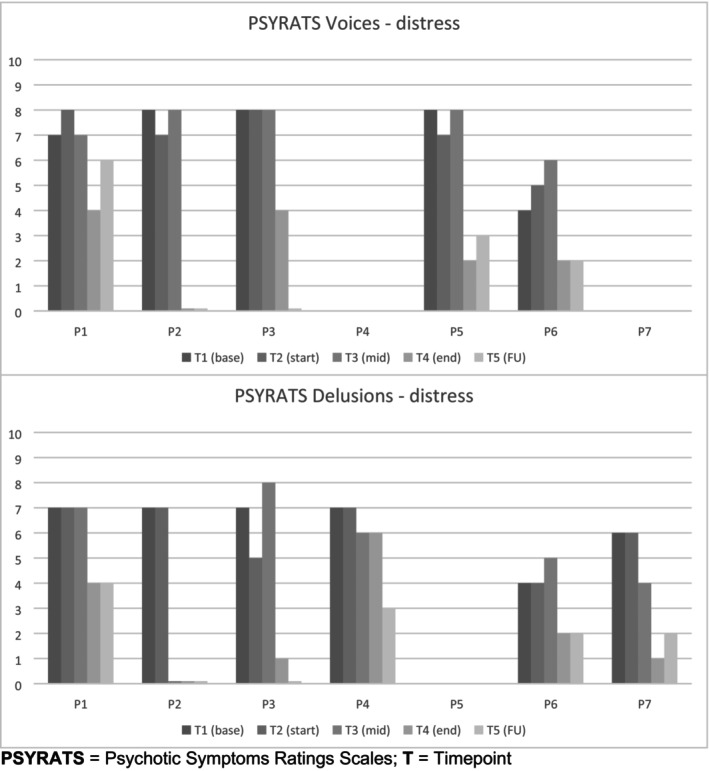
Changes in distressing voices and distressing delusions for each case. PSYRATS, Psychotic Symptoms Ratings Scales; *T*, timepoint.

Visual inspection of PSYRATS distress scores (for both voices and delusions) was of particular interest since distress associated with these experiences is a key CFTp target. A composite score of the two distress PSYRATS items (amount and intensity; 10‐point scale) was used to represent the changes in distress over time (Figure 3).

There are overall trends of distress reduction for all cases; however, the reduction patterns are slightly different for voices and delusions. For voices, distress stayed quite stable (high) for the first three timepoints (T1, T2 and T3) and then dropped for the final two (T4 and T5). This drop was greater for four cases (P2, P3, P5 and P6) than for one (P1). For delusions, there is a similar late reduction in distress for four cases (P1, P3, P6 and P6), but in one case (P2), there is a quick reduction by mid‐therapy (T3) and, for another case (P4), a slow reduction that does not occur until follow‐up (T5). These results suggest that CFTp is helpful for reducing distress, especially over the full (26 sessions) time period; however, no statistical analyses were performed on these data.

## DISCUSSION

CFTp was found to be feasible and acceptable to participants with distressing voices and/or delusions in community psychosis services. Only one of eight participants did not complete the intervention, and the seven who did engage with the full 26 sessions. This attrition rate (12.5%) is in keeping with the 16% reported in a meta‐analysis of CBT for psychosis (Lincoln et al., 2008). All seven provided a complete set of process and outcome measures, although eight (of 35) HRV recordings were later excluded due to technical issues. One participant opted out of weekly measures and audio‐recordings, and all opted out of video‐recordings.

The acceptability of this novel intervention is the most important finding of this study. CFTp techniques are unlikely to have been encountered by participants before in mental health services and may be in contradiction to how they have been accustomed to relating to their voices and delusions. For example, compassionate engaging with voices is in stark contrast to some styles of relating that are traditionally advanced by services. The CFTp approach [as with some other relational‐based therapies (Dellazizzo et al., 2022)] encourages a stance that is less about suppression and conflict, and more about curiosity and collaboration. Voices might be considered, for example, as having possible functions (e.g., protective) or holding information about emotional concerns (Heriot‐Maitland & Longden, 2022).

CFTp also requires participation in experiential practices, for example, role play, imagery and breathing/posture. That all seven participants engaged with such exercises for many months, amid distressing voices and delusions, are highly promising for future developments and research into CFTp. The one participant who did not complete therapy was not due to issues with the therapy content or procedure itself but to relapse in a pre‐existing alcohol condition. It was not clear whether the therapy had a role in triggering the relapse. This participant kept attending regular appointments with the same therapist (for clinical support, rather than CFTp), suggesting that the meetings were not aversive.

Key measures of process and outcome showed improvements, and most were clinically reliable and significant, compared to the baseline phase. These results suggest that, overall, CFTp successfully targeted the intended social mechanisms (e.g., reducing shame and self‐criticism, and increasing social safeness and self‐compassion), with the desired impact on outcomes (e.g., improving voices, delusions, depression and general well‐being). These effects are consistent with those reported for CFT in other clinical populations (Millard et al., 2023). The outcomes that showed reliable improvement for the most cases were depression (5/7), stress (5/7), psychological distress (5/7), anxiety (4/7) and voices (3/5).

At the group level, significant improvements were found in depression, stress, distress, voices and delusions. Whether changes in social processes preceded or occurred concurrently with these improved outcomes was inconclusive. However, there was evidence that self‐compassion (a process), delusions and depression (outcomes) took longer to change than other measures. There are parallels in the existing literature regarding timing of improvements; for example, Hickey et al. (2021) reported that self‐compassion and depression were slow to change for participants with psychosis attending an 8‐week mindfulness and compassion programme, only becoming significant at a 6‐week follow‐up (i.e., 14 weeks after group start). Khoury et al. (2015) also found that most scores (including depression and emotion regulation) only became significant at 3‐month follow‐up. In the current study, most improvements were maintained 6–8 weeks after CFTp, and it would be useful for future evaluations to track changes over extended follow‐up periods to see whether improvements strengthen over time.

The single‐case results showed that not all participants benefited to the same extent. Some had measures that did not change significantly and, on a small handful of occasions, changed significantly in the opposite direction. However, overall, it can be concluded that CFTp shows sufficient feasibility, impact and promise to warrant further testing in a pilot trial.

Overall, this study demonstrated good acceptability and feasibility of applying CFT psychoeducation, processes and interventions for people with psychosis.

### Single‐case variability in results

At the single‐case level, it was possible to distinguish variability across participants. Five (P2, P3, P4, P5 and P6) showed improvements across multiple outcomes, which accounted for the significant change detected at group level. Two participants (P1 and P7), however, improved on only one or two outcomes, with one or two that deteriorated. For example, both reported an increase in anxiety over the period to follow‐up (T2 → T5), despite several process measures improving over that same period, and their scores on voice‐ and delusion‐related distress reducing.

There were notable differences in anxiety and psychotic symptom results between the single‐case and group analysis. Anxiety improved reliably for four of the seven cases but did not reach significance at group level. This is likely due to anxiety deteriorating for one case (P1), affecting the group mean. The single‐case analysis found more cases with reliable improvement in voices (three of five) than delusions (two of six), whereas the significant improvement for delusions was of a higher magnitude than for voices at group level. Closer inspection of Table S3 shows that for the four cases whose delusions did not improve reliably, the RCIs were only marginally short of the reliable change threshold of −1.96, which may explain this discrepancy.

The weekly measures showed that, overall, CFTp produced an improvement in social safeness over therapy. The single‐case plots and analyses show that this effect was caused by three cases with significant improvements relative to baseline (P2, P3 and P4), one with a large improvement that was not significant relative to baseline (P5), and one with a steady, but small, improvement, not significant relative to baseline (P6). One case (P7) reported social safeness levels that improved less in therapy than in baseline, registering a negative trend. So, while it can be concluded that, overall, CFTp was producing improvements for most, there were still exceptions. This is common in mental health treatments, where it is unlikely that a treatment can help everyone, and especially so for people with more complex mental health difficulties.

### Limitations

The CFTp therapist was the intervention developer as well as the researcher, which has potential for evaluation bias. However, the research procedures were designed to minimize bias; for example, self‐report measures were used as far as possible and were adapted into electronic format so that participants could respond without researcher involvement. The only interviewer‐rated measure was the PSYRATS; however, the item ratings are based on the participants' own assessment of their symptoms with clear scoring guidelines.

Another limitation is that some measures did not have normative data from psychosis populations, so non‐psychosis data were used. Finally, technical difficulties led to missing data for HRV. Since this study was designed, technology has advanced so that more accessible, cheaper ‘wearable’, devices have become more widely available with improved accuracy, comparable to that of classic ECG (Georgiou et al., 2018).

### Conclusions and implications for future research

CFTp is a feasible and acceptable intervention for people with distressing psychotic experiences. There were enough indicators of effectiveness to warrant a pilot RCT, with the aim of establishing the design parameters for a full RCT. It will be important for other therapists (not the developer) to provide the interventions and for independent, blinded researchers to collect the measures. Most of the process and outcome measures used in this study were found to be feasible and sensitive to change, but one suggestion would be to focus on the PSYRATS distress scores, rather than total score, as a primary outcome measure (also recommended by Loizou et al., 2022). Visual inspection of PSYRATS distress (Figure 3) showed clear improvement trends in all seven cases, and this outcome is more closely aligned to the specific target of CFTp, which is concerned with shifting the threat‐based emotional relationship with psychotic symptoms. However, an important recommendation arising from the distress results is that CFTp should not be shortened, since for some, distress levels may go up first (13 sessions) before coming down considerably (26 sessions). Furthermore, key measures of self‐compassion, delusions and depression only started changing in the second half of therapy. Other symptoms of psychosis, such as negative symptoms, were not investigated in this study (nor in previous CFTp studies) and may be another useful avenue for future, follow‐up research.

To conclude, this study shows promise for CFTp as a treatment option for people experiencing distressing voices and/or delusions in the context of a psychosis‐related diagnosis and warrants progression to the more robust methodology of an RCT.

## AUTHOR CONTRIBUTIONS

**Charles Heriot‐Maitland:** Conceptualization; investigation; formal analysis; funding acquisition; writing – original draft; methodology; resources. **Andrew Gumley:** Supervision; writing – review and editing. **Til Wykes:** Funding acquisition; methodology; supervision; writing – review and editing. **Eleanor Longden:** Resources; supervision. **Chris Irons:** Resources; supervision. **Paul Gilbert:** Supervision; resources; writing – review and editing. **Emmanuelle Peters:** Supervision; methodology; funding acquisition; writing – review and editing.

## FUNDING INFORMATION

This work was supported by the Medical Research Council (CHM, grant number MR/L01677X/1).

## CONFLICT OF INTEREST STATEMENT

All authors declare no conflict of interest.

## Supporting information


Tables S1‐S6

## Data Availability

The data that support the findings of this study are available from the corresponding author upon reasonable request.
