# Supplementary material for: A case series study of compassion‐focused therapy for distressing experiences in psychosis
Source: Br J Clin Psychol. 2023 Aug 27;62(4):762–81. doi: 10.1111/bjc.12437 (PMC10946731; doi:10.1111/bjc.12437)
Supplement: Supplementary file 1 — Tables S1‐S6 [file BJC-62-762-s001.docx]

**Table S1.** Contents page of the CFTp Manual

**
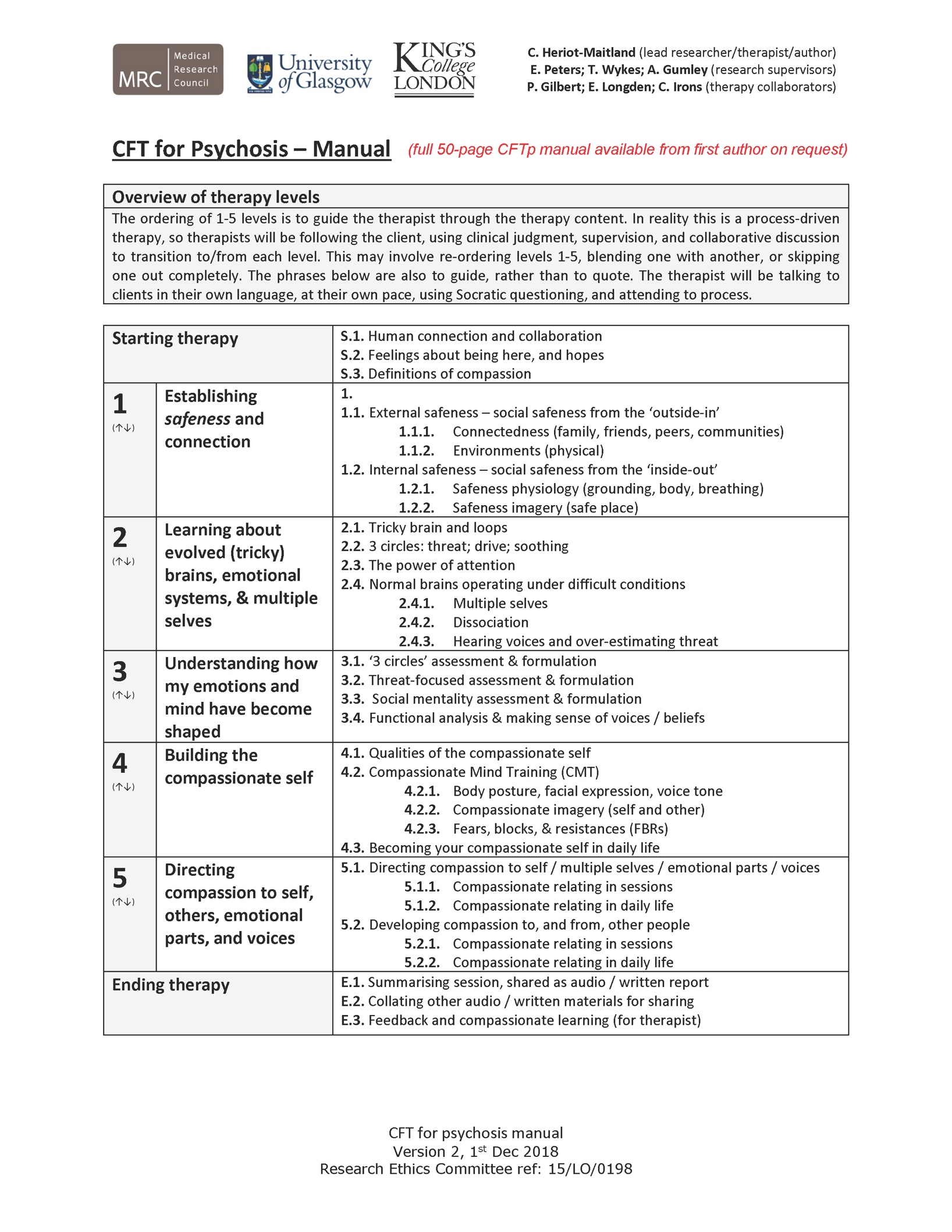
**

**Table S2.** Directions of reliable changes in process and outcome measures, showing only those that were significant against a Reliable Change Index (RCI). Arrows in **bold** signal the direction of improvement for that measure

| **Breakdown by single case** | | | | | | | | | |
| --- | --- | --- | --- | --- | --- | --- | --- | --- | --- |
| ***P1*** | **T1→T2** | **T2→T3** | **T2→T4** | **T2→T5** |  | **T1→T2** | **T2→T3** | **T2→T4** | **T2→T5** |
| *Process measures (RCI)* | | |  |  | *Outcome measures (RCI)* | | |  |  |
| **SocC^1^** | - | - | - | - | **PSYRATS-V** | - | - | - | - |
| **FSCSR-Inad** | - | - | - | - | **PSYRATS-D** | - | - | **↓** | - |
| **FSCSR-Reas** | - | **↑** | **↑** | - | **DASS-Dep** | - | **↓** | - | **↓** |
| **FSCSR-Hate** | - | **↓** | **↓** | - | **DASS-Anx** | ↓ | **↑** | **↑** | **↑** |
| **OAS** | - | - | - | **↓** | **DASS-Str** | ↑ | **↓** | **↓** | **↓** |
| **SCS-SF** | - | - | **↑** | - | **CORE** | - | - | - | - |
| **PBIQ-R** | - | **↓** | **↓** | **↓** | **DES-II** | - | - | ↑ | - |
| **RMSSD (ms)** | - | **↑** | md^2^ | - |  |  |  |  |  |
| ***P2*** | **T1→T2** | **T2→T3** | **T2→T4** | **T2→T5** |  | **T1→T2** | **T2→T3** | **T2→T4** | **T2→T5** |
| *Process measures (RCI)* | | |  |  | *Outcome measures (RCI)* | | |  |  |
| **SocC** | - | **↑** | **↑** | **↑** | **PSYRATS-V** | - | - | **↓** | **↓** |
| **FSCSR-Inad** | - | - | - | **↓** | **PSYRATS-D** | - | **↓** | **↓** | **↓** |
| **FSCSR-Reas** | - | **↑** | **↑** | **↑** | **DASS-Dep** | - | - | **↓** | **↓** |
| **FSCSR-Hate** | - | **↓** | **↓** | **↓** | **DASS-Anx** | - | **↓** | **↓** | **↓** |
| **OAS** | **↓** | **↓** | **↓** | **↓** | **DASS-Str** | ↑ | **↓** | **↓** | **↓** |
| **SCS-SF** | - | - | **↑** | **↑** | **CORE** | - | **↓** | **↓** | **↓** |
| **PBIQ-R** | - | ↑ | **↓** | **↓** | **DES-II** | - | **↓** | **↓** | **↓** |
| **RMSSD (ms)** | **↑** | md | md | ↓ |  |  |  |  |  |
| ***P3*** | **T1→T2** | **T2→T3** | **T2→T4** | **T2→T5** |  | **T1→T2** | **T2→T3** | **T2→T4** | **T2→T5** |
| *Process measures (RCI)* | | |  |  | *Outcome measures (RCI)* | | |  |  |
| **SocC** | - | - | - | - | **PSYRATS-V** | - | - | - | **↓** |
| **FSCSR-Inad** | - | - | **↓** | - | **PSYRATS-D** | - | - | - | - |
| **FSCSR-Reas** | - | - | - | - | **DASS-Dep** | - | **↓** | **↓** | **↓** |
| **FSCSR-Hate** | - | **↓** | - | - | **DASS-Anx** | **↓** | **↓** | - | - |
| **OAS** | - | - | - | - | **DASS-Str** | - | - | **↓** | - |
| **SCS-SF** | - | **↑** | **↑** | **↑** | **CORE** | - | **↓** | **↓** | **↓** |
| **PBIQ-R** | - | **↓** | **↓** | **↓** | **DES-II** | - | - | - | **↓** |
| **RMSSD (ms)** | ↓ | - | **↑** | - |  |  |  |  |  |
| ***P4*** | **T1→T2** | **T2→T3** | **T2→T4** | **T2→T5** |  | **T1→T2** | **T2→T3** | **T2→T4** | **T2→T5** |
| *Process measures (RCI)* | | |  |  | *Outcome measures (RCI)* | | |  |  |
| **SocC** | ↓ | **↑** | **↑** | **↑** | **PSYRATS-V** | nv^3^ | nv | nv | nv |
| **FSCSR-Inad** | - | - | **↓** | **↓** | **PSYRATS-D** | - | - | - | - |
| **FSCSR-Reas** | ↓ | **↑** | **↑** | **↑** | **DASS-Dep** | - | **↓** | **↓** | **↓** |
| **FSCSR-Hate** | - | - | **↓** | **↓** | **DASS-Anx** | - | **↓** | **↓** | **↓** |
| **OAS** | **↓** | - | - | - | **DASS-Str** | - | **↓** | **↓** | **↓** |
| **SCS-SF** | - | - | - | **↑** | **CORE** | ↑ | **↓** | **↓** | **↓** |
| **PBIQ-R** | - | **↓** | **↓** | **↓** | **DES-II** | - | - | - | - |
| **RMSSD (ms)** | md | **↑** | - | md |  |  |  |  |  |
| ***P5*** | **T1→T2** | **T2→T3** | **T2→T4** | **T2→T5** |  | **T1→T2** | **T2→T3** | **T2→T4** | **T2→T5** |
| *Process measures (RCI)* | | | | | *Outcome measures (RCI)* | | | | |
| **SocC** | ↓ | **↑** | **↑** | **↑** | **PSYRATS-V** | - | - | **↓** | **↓** |
| **FSCSR-Inad** | - | **↓** | **↓** | **↓** | **PSYRATS-D** | nd^4^ | nd | nd | nd |
| **FSCSR-Reas** | **↑** | ↓ | - | ↓ | **DASS-Dep** | - | **↓** | **↓** | **↓** |
| **FSCSR-Hate** | - | **↓** | **↓** | **↓** | **DASS-Anx** | ↑ | **↓** | **↓** | **↓** |
| **OAS** | - | **↓** | **↓** | **↓** | **DASS-Str** | - | **↓** | **↓** | **↓** |
| **SCS-SF** | - | - | **↑** | - | **CORE** | - | **↓** | **↓** | **↓** |
| **PBIQ-R** | - | **↓** | **↓** | **↓** | **DES-II** | - | **↓** | **↓** | **↓** |
| **RMSSD (ms)** | md | - | - | - |  |  |  |  |  |
| ***P6*** | **T1→T2** | **T2→T3** | **T2→T4** | **T2→T5** |  | **T1→T2** | **T2→T3** | **T2→T4** | **T2→T5** |
| *Process measures (RCI)* | | |  |  | *Outcome measures (RCI)* | | |  |  |
| **SocC** | - | - | **↑** | **↑** | **PSYRATS-V** | - | - | **↓** | **↓** |
| **FSCSR-Inad** | - | **↓** | **↓** | **↓** | **PSYRATS-D** | - | - | - | - |
| **FSCSR-Reas** | - | - | - | - | **DASS-Dep** | **↓** | ↑ | - | - |
| **FSCSR-Hate** | - | - | - | - | **DASS-Anx** | - | - | - | - |
| **OAS** | - | - | **↓** | **↓** | **DASS-Str** | **↓** | ↑ | - | - |
| **SCS-SF** | - | - | - | - | **CORE** | - | - | **↓** | **↓** |
| **PBIQ-R** | - | **↓** | **↓** | **↓** | **DES-II** | - | **↓** | - | **↓** |
| **RMSSD (ms)** | - | - | - | - |  |  |  |  |  |
| ***P7*** | **T1→T2** | **T2→T3** | **T2→T4** | **T2→T5** |  | **T1→T2** | **T2→T3** | **T2→T4** | **T2→T5** |
| *Process measures (RCI)* | | |  |  | *Outcome measures (RCI)* | | |  |  |
| **SocC** | - | - | - | - | **PSYRATS-V** | nv | nv | nv | nv |
| **FSCSR-Inad** | **↓** | - | - | **↓** | **PSYRATS-D** | - | - | - | - |
| **FSCSR-Reas** | - | ↓ | - | - | **DASS-Dep** | - | **↓** | **↓** | - |
| **FSCSR-Hate** | ↑ | **↓** | **↓** | **↓** | **DASS-Anx** | - | - | **↓** | ↑ |
| **OAS** | - | **↓** | - | **↓** | **DASS-Str** | - | **↓** | - | ↑ |
| **SCS-SF** | **↑** | ↓ | - | - | **CORE** | - | - | - | - |
| **PBIQ-R** | - | **↓** | - | **↓** | **DES-II** | - | - | - | - |
| **RMSSD (ms)** | md | ↓ | **↑** | md |  |  |  |  |  |

**^1^SocC** = Social Comparison Scale; **FSCSR** = Forms of Self-Criticising/Attacking and Self-Reassuring Scale (**Inad** = Inadequate-self, **Reas** = Self-reassurance, **Hate** = Hated-self); **OAS** = Other as Shamer Scale; **SCS-SF** = Self-Compassion Scale Short-Form; **PBIQ-R** = Personal Beliefs about Illness Questionnaire-Revised; **RMSSD (ms)** = Root Mean Square of Successive Differences (milliseconds); **PSYRATS** = Psychotic Symptoms Ratings Scales (**V** = Voices, **D** = Delusions); **DASS** = Depression Anxiety and Stress Scales (**Dep** = Depression, **Anx** = Anxiety, **Str** = Stress); **CORE** = Clinical Outcomes in Routine Evaluation; **DES-II** = Revised Dissociative Experiences Scale.

^2^md = missing data; ^3^nv = no voices; ^4^nd = no delusions

**Table S3.** Reliable Change Indices for single-case-level changes across phases – full table

|  | **Baseline phase**  **(T1→T2)** | | **1st half phase**  **(T2→T3)** | | **Intervention phase**  **(T2→T4)** | | **Follow-up**  **(T2→T5)** | |
| --- | --- | --- | --- | --- | --- | --- | --- | --- |
|  | *Pt* | *RCI* | *Pt* | *RCI* | *Pt* | *RCI* | *Pt* | *RCI* |
| *Outcome measures* | | | | | | | | |
| **PSYRATS-V** | 1  2  3  4  5  6  7 | .18  .00  -.18  *No voices*  -.89  .18  *No voices* | 1  2  3  4  5  6  7 | -.72  -1.61  -.18  *No voices*  -.89  -.72  *No voices* | 1  2  3  4  5  6  7 | -1.61  -5.01*  -1.79  *No voices*  -3.04*  -2.50*  *No voices* | 1  2  3  4  5  6  7 | -1.61  -5.01*  -2.68*  *No voices*  -3.04*  -2.33*  *No voices* |
|  | No RC = 5 | | No RC = 5 | | No RC = 2  ↓ = 3 | | No RC = 1  ↓ = 4 | |
| **PSYRATS-D** | 1  2  3  4  5  6  7 | .00  .00  -.82  .00  *No delusions*  .00  .00 | 1  2  3  4  5  6  7 | .00  -3.53*  1.36  -.54  *No delusions*  -.27  -1.36 | 1  2  3  4  5  6  7 | -2.45*  -3.53*  -1.63  -1.09  *No delusions*  -1.63  -1.90 | 1  2  3  4  5  6  7 | -1.63  -3.53*  -1.90  -1.63  *No delusions*  -1.63  -1.90 |
|  | No RC = 6 | | No RC = 5  ↓ = 1 | | No RC = 4  ↓ = 2 | | No RC = 5  ↓ = 1 | |
| **DASS-Dep** | 1  2  3  4  5  6  7 | 1.83  -1.83  -.92  .92  .92  -2.75*  .92 | 1  2  3  4  5  6  7 | -3.67*  -.92  -8.26*  -5.50*  -10.09*  3.67*  -4.59* | 1  2  3  4  5  6  7 | -1.83  -8.26*  -11.01*  -8.26*  -11.01*  1.83  -4.59* | 1  2  3  4  5  6  7 | -3.67*  -12.84*  -11.93*  -8.26*  -13.76*  1.83  .92 |
|  | No RC = 6  ↓ = 1 | | ↑ = 1  No RC = 1  ↓ = 5 | | No RC = 2  ↓ = 5 | | No RC = 2  ↓ = 5 | |
| **DASS-Anx** | 1  2  3  4  5  6  7 | -7.69*  .96  -2.88*  1.92  2.88*  .96  1.92 | 1  2  3  4  5  6  7 | 4.81*  -4.81*  -2.88*  -5.77*  -6.73*  .00  -1.92 | 1  2  3  4  5  6  7 | 5.77*  -5.77*  -.96  -9.62*  -9.62*  -.96  -2.88* | 1  2  3  4  5  6  7 | 4.81*  -9.62*  -1.92  -7.69*  -15.38*  .00  5.77* |
|  | ↑ = 1  No RC = 4  ↓ = 2 | | ↑ = 1  No RC = 2  ↓ = 4 | | ↑ = 1  No RC = 2  ↓ = 4 | | ↑ = 2  No RC = 2  ↓ = 3 | |
| **DASS-Str** | 1  2  3  4  5  6  7 | 3.62*  3.62*  .00  .90  .00  -3.62*  -1.81 | 1  2  3  4  5  6  7 | -5.43*  -5.43*  -.90  -6.33*  -9.95*  2.71*  -3.62* | 1  2  3  4  5  6  7 | -7.24*  -6.33*  -2.71*  -10.86*  -10.86*  -.90  -1.81 | 1  2  3  4  5  6  7 | -9.05*  -9.05*  -1.81  -9.05*  -13.57*  -1.81  2.71* |
|  | ↑ = 2  No RC = 4  ↓ = 1 | | ↑ = 1  No RC = 1  ↓ = 5 | | No RC = 2  ↓ = 5 | | ↑ = 1  No RC = 2  ↓ = 4 | |
| **CORE** | 1  2  3  4  5  6  7 | -1.70  .79  -.45  2.38*  -.11  -1.47  1.13 | 1  2  3  4  5  6  7 | -.90  -4.64*  -3.96*  -3.62*  -5.77*  1.13  -1.24 | 1  2  3  4  5  6  7 | -.68  -6.22*  -4.07*  -4.41*  -7.35*  -4.75*  -1.36 | 1  2  3  4  5  6  7 | -1.92  -8.37*  -3.73*  -3.96*  -9.05*  -4.41*  -1.02 |
|  | ↑ = 1  No RC = 6 | | No RC = 3  ↓ = 4 | | No RC = 2  ↓ = 5 | | No RC = 2  ↓ = 5 | |
| **DES-II** | 1  2  3  4  5  6  7 | -1.14  .91  .38  1.29  .38  1.29  -.84 | 1  2  3  4  5  6  7 | .84  -3.05*  -.61  .38  -7.61*  -3.05*  .15 | 1  2  3  4  5  6  7 | 2.28*  -3.12*  -1.29  -1.37  -9.06*  -1.83  .84 | 1  2  3  4  5  6  7 | .61  -2.59*  -1.98*  -1.22  -9.67*  -2.44*  .23 |
|  | No RC = 7 | | No RC = 4  ↓ = 3 | | ↑ = 1  No RC = 4  ↓ = 2 | | No RC = 3  ↓ = 4 | |
| *Process measures* | | | | | | | | |
| **SocC** | 1  2  3  4  5  6  7 | -1.14  .00  1.00  -3.56*  -2.42*  .00  .28 | 1  2  3  4  5  6  7 | .85  4.70*  -.14  3.85*  6.13*  1.14  1.14 | 1  2  3  4  5  6  7 | 1.71  3.99*  1.42  5.41*  10.54*  6.98*  1.00 | 1  2  3  4  5  6  7 | .43  2.56*  -.14  6.27*  8.97*  5.98*  -1.00 |
|  | No RC = 5  ↓ = 2 | | ↑ = 3  No RC = 4 | | ↑ = 4  No RC = 3 | | ↑ = 4  No RC = 3 | |
| **FSCSR-Inad** | 1  2  3  4  5  6  7 | .00  -.78  -1.57  1.04  -.26  1.31  -3.13* | 1  2  3  4  5  6  7 | -.78  -1.57  .52  -1.83  -4.18*  -2.35*  -.78 | 1  2  3  4  5  6  7 | -.52  -1.31  -2.35*  -3.13*  -6.01*  -2.35*  .00 | 1  2  3  4  5  6  7 | .78  -5.74*  -1.04  -2.35*  -7.05*  -4.70*  -3.39* |
|  | No RC = 6  ↓ = 1 | | No RC = 5  ↓ = 2 | | No RC = 3  ↓ = 4 | | No RC = 2  ↓ = 5 | |
| **FSCSR-Reas** | 1  2  3  4  5  6  7 | .31  1.25  .31  -3.74*  5.61*  -1.87  -.93 | 1  2  3  4  5  6  7 | 4.36*  2.18*  .00  3.12*  -3.74*  -.62  -2.49* | 1  2  3  4  5  6  7 | 2.18*  3.12*  1.56  3.43*  -1.56  1.56  .00 | 1  2  3  4  5  6  7 | .62  5.61*  -.62  2.49*  -2.49*  .62  -1.87 |
|  | ↑ = 1  No RC = 5  ↓ = 1 | | ↑ = 3  No RC = 2  ↓ = 2 | | ↑ = 3  No RC = 4 | | ↑ = 2  No RC = 4  ↓ = 1 | |
| **FSCSR-Hate** | 1  2  3  4  5  6  7 | -.73  .00  .00  .73  1.09  1.45  3.27* | 1  2  3  4  5  6  7 | -4.00*  -4.36*  -2.18*  -1.09  -3.64*  -1.09  -2.55* | 1  2  3  4  5  6  7 | -2.91*  -4.73*  -1.82  -2.18*  -4.73*  -1.09  -3.64* | 1  2  3  4  5  6  7 | .00  -5.45*  -1.45  -2.91*  -4.73*  -1.09  -4.36* |
|  | ↑ = 1  No RC = 6 | | No RC = 2  ↓ = 5 | | No RC = 2  ↓ = 5 | | No RC = 3  ↓ = 4 | |
| **OAS** | 1  2  3  4  5  6  7 | .61  -2.04*  -1.64  -4.70*  1.02  1.02  -.41 | 1  2  3  4  5  6  7 | .61  -3.27*  -.41  -1.23  -8.38*  -1.64  -4.09* | 1  2  3  4  5  6  7 | -1.84  -6.54*  -1.23  -.82  -11.66*  -4.91*  -1.23 | 1  2  3  4  5  6  7 | -2.86*  -7.98*  -.20  -1.64  -12.68*  -5.73*  -3.68* |
|  | No RC = 5  ↓ = 2 | | No RC = 4  ↓ = 3 | | No RC = 4  ↓ = 3 | | No RC = 2  ↓ = 5 | |
| **SCS-SF** | 1  2  3  4  5  6  7 | .40  1.40  -.40  -.60  -.40  .20  2.00* | 1  2  3  4  5  6  7 | .60  1.60  2.20*  1.40  .20  -.80  -2.20* | 1  2  3  4  5  6  7 | 2.60*  2.40*  2.20*  1.60  2.00*  .40  -1.20 | 1  2  3  4  5  6  7 | 1.20  4.40*  2.60*  2.20*  1.40  .80  -.80 |
|  | ↑ = 1  No RC = 6 | | ↑ = 1  No RC = 5  ↓ = 1 | | ↑ = 4  No RC = 3 | | ↑ = 3  No RC = 4 | |
| **PBIQ-R** | 1  2  3  4  5  6  7 | -.71  -.88  -.18  .35  -.35  1.42  .71 | 1  2  3  4  5  6  7 | -2.48*  -1.95  -2.83*  -3.19*  -7.43*  -2.12*  -2.12* | 1  2  3  4  5  6  7 | -3.01*  -6.37*  -4.07*  -3.36*  -9.20*  -5.66*  -.18 | 1  2  3  4  5  6  7 | -3.54*  -3.72*  -2.30*  -3.19*  -9.56*  -3.54*  -2.65* |
|  | No RC = 7 | | No RC = 1  ↓ = 6 | | No RC = 1  ↓ = 6 | | ↓ = 7 | |
| **RMDDS (ms)** | 1  2  3  4  5  6  7 | -.20  3.13*  -2.53*  -.30 | 1  2  3  4  5  6  7 | 2.83*  1.31  2.93*  1.21  -1.11  -4.24* | 1  2  3  4  5  6  7 | 3.23*  1.01  1.11  -.91  13.64* | 1  2  3  4  5  6  7 | 1.11  -3.84*  .61  -.51  -1.31 |
|  | ↑ = 1  No RC = 2  ↓ = 1 | | ↑ = 2  No RC = 3  ↓ = 1 | | ↑ = 2  No RC = 3 | | No RC = 4  ↓ = 1 | |

*p<.05

**Table S4.** Means and standard deviations for outcome and process measures at five assessment points

|  | **Start baseline**  **(T1)** | | **Start therapy**  **(T2)** | | **Mid therapy**  **(T3)** | | **End Therapy**  **(T4)** | | **Follow up**  **(T5)** | |
| --- | --- | --- | --- | --- | --- | --- | --- | --- | --- | --- |
|  | *Mean* | *(SD)* | *Mean* | *(SD)* | *Mean* | *(SD)* | *Mean* | *(SD)* | *Mean* | *(SD)* |
| *Outcome measures* | | | | |  |  |  |  |  |  |
| **PSYRATS-V^1^** | 22.71 | (16.03) | 22.14 | (15.33) | 18.86 | (13.64) | 11.00 | (10.91) | 10.43 | (10.03) |
| **PSYRATS-D** | 14.71 | (6.99) | 14.29 | (6.78) | 12.00 | (8.85) | 7.86 | (5.61) | 7.86 | (5.76) |
| **DASS-Dep** | 28.00 | (4.76) | 27.71 | (5.22) | 18.57 | (6.60) | 14.29 | (6.87) | 12.86 | (10.45) |
| **DASS-Anx** | 21.14 | (7.10) | 20.57 | (9.22) | 15.43 | (8.85) | 13.43 | (8.77) | 13.43 | (11.98) |
| **DASS-Str** | 27.43 | (11.53) | 28.29 | (10.67) | 19.14 | (10.67) | 15.43 | (9.36) | 15.14 | (11.65) |
| **CORE** | 2.24 | (0.51) | 2.26 | (0.47) | 1.56 | (0.69) | 1.19 | (0.62) | 1.06 | (0.71) |
| **DES-II** | 23.27 | (18.61) | 24.80 | (19.11) | 16.12 | (13.07) | 15.71 | (17.13) | 13.37 | (15.21) |
| *Process measures* | | | |  |  |  |  |  |  |  |
| **SocC** | 35.29 | (9.05) | 29.43 | (16.87) | 47.14 | (15.48) | 60.57 | (16.69) | 52.57 | (16.13) |
| **FSCSR-Inad** | 29.71 | (4.79) | 27.86 | (4.95) | 21.86 | (3.89) | 19.29 | (5.65) | 15.00 | (10.38) |
| **FSCSR-Reas** | 13.57 | (7.89) | 14.00 | (8.94) | 15.29 | (4.03) | 18.71 | (5.79) | 16.00 | (9.31) |
| **FSCSR-Hate** | 10.00 | (5.42) | 12.29 | (3.25) | 4.86 | (2.12) | 4.00 | (2.38) | 4.43 | (5.44) |
| **OAS** | 48.71 | (15.99) | 44.43 | (15.74) | 31.57 | (13.79) | 24.71 | (10.64) | 20.14 | (13.18) |
| **SCS-SF** | 28.29 | (6.80) | 30.14 | (6.44) | 32.29 | (7.59) | 37.29 | (7.34) | 38.57 | (9.91) |
| **PBIQ-R** | 68.43 | (9.95) | 68.71 | (6.80) | 50.86 | (9.49) | 43.00 | (12.25) | 45.71 | (12.63) |
| **RMSSD (ms)** | 0.05 | (0.02) | 0.04 | (0.03) | 0.04 | (0.03) | 0.06 | (0.07) | 0.04 | (0.03) |

^1^All measures and timepoints are n=7, except for **PSYRATS-V** (n=5 for all timepoints), **PSYRATS-D** (n=6 for all timepoints), and **RMSSD** (n=4 for T1, n=7 for T2, n=6 for T3, n=5 for T4, n=5 for T5). For **RMSSD**, all n=7 provided data at all timepoints, however some were excluded due to incorrect software setup (4 occasions) and poor ECG connection (4 occasions).

**Table S5.** Summary of Tau analysis for sessional process measure (SSPS)

|  | **Participant** | **Tau** | ***SD*_Tau_** | ***p*-value** | **85% conf. interval** |
| --- | --- | --- | --- | --- | --- |
|  |  |  |  |  |  |
| **Baseline**  **(baseline trend)** | P2 “Greg” | .24 | .32 | .45 | -.22, .70 |
|  | P3 “Thomas” | .00 | .49 | 1.00 | -.71, .71 |
|  | P4 “Tosin” | .67 | .64 | .30 | -.25, 1.00 |
|  | P5 “Charmaine” | .33 | .64 | .60 | -.59, 1.00 |
|  | P6 “Amanda” | .40 | .41 | .33 | -.19, .99 |
|  | P7 “Gareth” | .14 | .32 | .65 | -.31, .60 |
| **Intervention (phase change)** | P2 “Greg” | .95 | .25 | **<.001** | .59, 1.00 |
|  | P3 “Thomas” | .98 | .32 | **<.001** | .52, 1.00 |
|  | P4 “Tosin” | 1.00 | .36 | **<.001** | .48, 1.00 |
|  | P5 “Charmaine” | .37 | .36 | .30 | -.14, .89 |
|  | P6 “Amanda” | .45 | .28 | .11 | .04, .86 |
|  | P7 “Gareth” | -.63 | .25 | **.01** | -.99, -.27 |
| **Combined**  **(phase change)** | P2+P3+P4+P5+P6+P7 | .48 |  | **<.001** | .30, .66 |

Significant results in **bold**

**Table S6.** Summary of Tau analysis for sessional outcome measure (Dissociation)

|  | **Participant** | **Tau** | ***SD*_Tau_** | ***p*-value** | **85% conf. interval** |
| --- | --- | --- | --- | --- | --- |
|  |  |  |  |  |  |
| **Baseline**  **(baseline trend)** | P2 “Greg” | .38 | .32 | .23 | -.08, .84 |
|  | P3 “Thomas” | .00 | .49 | 1.00 | -.71, .71 |
|  | P4 “Tosin” | .00 | .64 | 1.00 | -.92, .92 |
|  | P5 “Charmaine” | .00 | .64 | 1.00 | -.92, .92 |
|  | P6 “Amanda” | .00 | .41 | 1.00 | -.59, .59 |
|  | P7 “Gareth” | -.05 | .32 | .88 | -.50, .41 |
| **Intervention (phase change)** | P2 “Greg” | -.35 | .25 | .16 | -.71, .01 |
|  | P3 “Thomas” | -.06 | .32 | .84 | -.52, .39 |
|  | P4 “Tosin” | -.96 | .36 | **<.001** | -1.00, -.44 |
|  | P5 “Charmaine” | -.81 | .36 | **.02** | -1.00, -.30 |
|  | P6 “Amanda” | .66 | .28 | **.02** | .26, 1.00 |
|  | P7 “Gareth” | .18 | .25 | .47 | -.18, .54 |
| **Combined**  **(phase change)** | P2+P3+P4+P5+P6+P7 | -0.17 |  | .17 | -.35, .01 |

Significant results in **bold**
